# Polyglutamine- and Temperature-Dependent Conformational Rigidity in Mutant Huntingtin Revealed by Immunoassays and Circular Dichroism Spectroscopy

**DOI:** 10.1371/journal.pone.0112262

**Published:** 2014-12-02

**Authors:** Valentina Fodale, Natalie C. Kegulian, Margherita Verani, Cristina Cariulo, Lucia Azzollini, Lara Petricca, Manuel Daldin, Roberto Boggio, Alessandro Padova, Rainer Kuhn, Robert Pacifici, Douglas Macdonald, Ryan C. Schoenfeld, Hyunsun Park, J. Mario Isas, Ralf Langen, Andreas Weiss, Andrea Caricasole

**Affiliations:** 1 IRBM Promidis, Pomezia, Rome, Italy; 2 Department of Biochemistry and Molecular Biology, Zilkha Neurogenetic Institute, Keck School of Medicine, University of Southern California, Los Angeles, California, United States of America; 3 CHDI Management/CHDI Foundation, Los Angeles, California, United States of America; University of Pittsburgh School of Medicine, United States of America

## Abstract

**Background:**

In Huntington's disease, expansion of a CAG triplet repeat occurs in exon 1 of the huntingtin gene (*HTT*), resulting in a protein bearing>35 polyglutamine residues whose N-terminal fragments display a high propensity to misfold and aggregate. Recent data demonstrate that polyglutamine expansion results in conformational changes in the huntingtin protein (HTT), which likely influence its biological and biophysical properties. Developing assays to characterize and measure these conformational changes in isolated proteins and biological samples would advance the testing of novel therapeutic approaches aimed at correcting mutant HTT misfolding. Time-resolved Förster energy transfer (TR-FRET)-based assays represent high-throughput, homogeneous, sensitive immunoassays widely employed for the quantification of proteins of interest. TR-FRET is extremely sensitive to small distances and can therefore provide conformational information based on detection of exposure and relative position of epitopes present on the target protein as recognized by selective antibodies. We have previously reported TR-FRET assays to quantify HTT proteins based on the use of antibodies specific for different amino-terminal HTT epitopes. Here, we investigate the possibility of interrogating HTT protein conformation using these assays.

**Methodology/Principal Findings:**

By performing TR-FRET measurements on the same samples (purified recombinant proteins or lysates from cells expressing HTT fragments or full length protein) at different temperatures, we have discovered a temperature-dependent, reversible, polyglutamine-dependent conformational change of wild type and expanded mutant HTT proteins. Circular dichroism spectroscopy confirms the temperature and polyglutamine-dependent change in HTT structure, revealing an effect of polyglutamine length and of temperature on the alpha-helical content of the protein.

**Conclusions/Significance:**

The temperature- and polyglutamine-dependent effects observed with TR-FRET on HTT proteins represent a simple, scalable, quantitative and sensitive assay to identify genetic and pharmacological modulators of mutant HTT conformation, and potentially to assess the relevance of conformational changes during onset and progression of Huntington's disease.

## Introduction

Huntington's disease (HD) is a genetic neurodegenerative condition resulting from the expansion of a polyglutamine (polyQ) encoding region within exon 1 of the huntingtin gene (*HTT*; [1]). Mutant huntingtin protein (mHTT) can be processed to generate amino-terminal (N-terminal) fragments which, owing to the polyQ expansion, misfold and aggregate in the cytoplasm and nucleus of distinct cell types in the CNS and the periphery [2]–[5]. HD is characterized by the deposition of these insoluble macroaggregates, particularly in striatal and cortical neurons and involving the formation of poorly characterized intermediate states, including soluble oligomeric forms [6]–[8]. Although the relationship between macroaggregates and neurotoxicity is still unclear [9]–[12], the correlation between disease/phenotype status and aggregate load found in postmortem brains from HD patients and animal models highlights the importance of investigating their formation and properties [9], [13]–[15]. mHTT fragment aggregation is critically influenced by sequences preceding (amino-terminal 17 residues; N17) and following (proline-rich region; PRR) the polyQ stretch, as well as by post-translational modifications within exon 1 [16]–[23]. In fact, structural studies have revealed significant structural flexibility of the polyQ region [24], [25]. Recent evidence indicates that expansion of the polyQ region in mHTT results in decreased conformational flexibility of the polyQ region and decreased interactions between and/or relative positioning of flanking sequences [26]. In this study we probed the conformational flexibility of HTT taking advantage of robust, sensitive, rapid time-resolved Förster resonance energy transfer (TR-FRET) immunoassays we previously developed to quantify different HTT conformers [13]. TR-FRET immunoassay detection is based on the labeling of an antibody pair with a rare earth ion fluorophore donor and an acceptor fluorophore, thereby producing a specific TR-FRET signal when the donor and acceptor labeled antibodies bind to their antigen simultaneously. mHTT protein can be measured and differentiated from wild type HTT using MW1, a polyQ-specific antibody displaying higher avidity for expanded (mutant) polyQ HTT [27]. By performing TR-FRET measurements on recombinant, purified amino-terminal fragments comprising the first 548 residues of wild type HTT (N548 HTT) and mHTT at different temperatures, we have discovered a temperature-dependent, reversible, polyQ-dependent conformational change of wild type and mutant HTT proteins. We have confirmed the conformational nature of the effect by performing circular dichroism spectroscopy on recombinant HTT proteins at different temperatures, revealing striking temperature- and polyQ- dependent changes in the alpha-helical structure of HTT's N-terminal region. By investigating the temperature- and polyQ-dependent variation in TR-FRET signal using antibody pairs targeted at different N-terminal epitopes in HTT recombinant proteins and in lysates from cells transfected with HTT cDNAs of different lengths (exon 1, N548 and full length), we observed a consistent dependence of the effect on detection of the polyQ region, irrespective of protein length. This novel assay can potentially be employed to investigate HTT conformation in samples of different origins (preclinical and clinical) as well as to identify modulators of HTT conformation.

## Results

### HTT conformation is reversibly affected by temperature as measured by TR-FRET immunoassays

**Figure 1:**
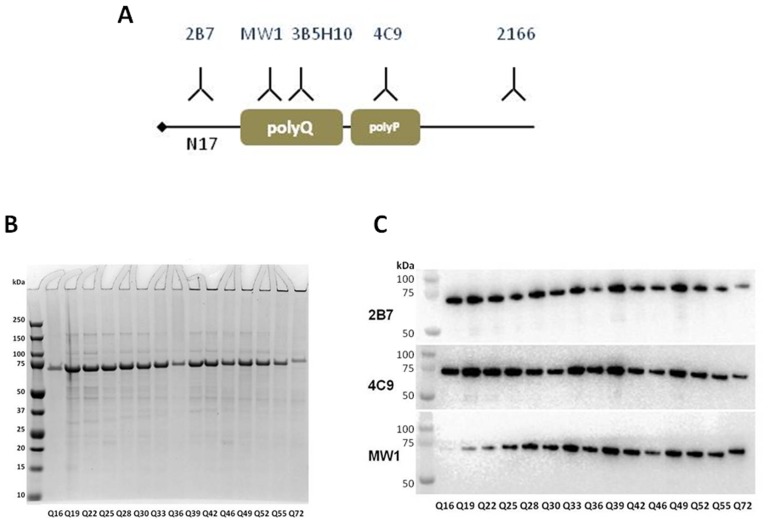
Epitopes of antibodies and N548 human recombinant HTT (hrHTT) proteins used in the study. A. Schematic positioning of epitopes for antibodies employed in these studies. 2B7 (recognizing the N17 domain), MW1 and 3B5H10 (specific for the polyQ region), 4C9 (recognizing an epitope within the PRR region) and 2166 (specific for an epitope around residue K444). B. Coomassie gel staining of the N548 hrHTT proteins, 5 µg of each protein bearing the indicated polyQ repeat length was loaded on the gel. C. Western blot of N548 hrHTT proteins employed in the TR-FRET analyses, probed with 2B7, MW1 and an antibody specific for a domain within the PRR region (4C9). 0.25 µg of protein bearing the indicated polyQ repeat length.

**Figure 2:**
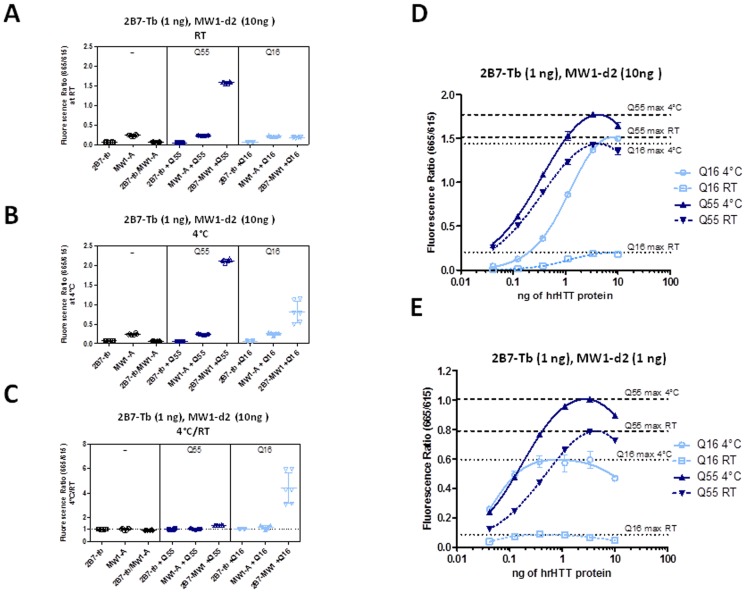
TR-FRET signals obtained using 2B7 and MW1 antibodies on hrHTT N548 wild type (Q16) and mutant (Q55) proteins are influenced by temperature and polyQ length. A-B. Fluorescence signal, expressed as fluorescence ratio (665 nm/615 nm), obtained with the antibodies (2B7-Tb and MW1-d2) tested individually or in combination with N548 hrHTT proteins (Q16 or Q55), at RT (A) and 4°C (B). C. Ratio between the fluorescence signals observed at the two temperatures (in A and B, 4°C/RT). D. TR-FRET fluorescence signals from a dilution curve of N548 hrHTT proteins obtained using 1 ng of 2B7-Tb and 10 ng of MW1-d2 antibody combination at RT or 4°C. E. Same as D, TR-FRET performed using 1 ng of 2B7-Tb and 1 ng of MW1-d2 antibody combination. Results from representative experiments are shown.

**Figure 3:**
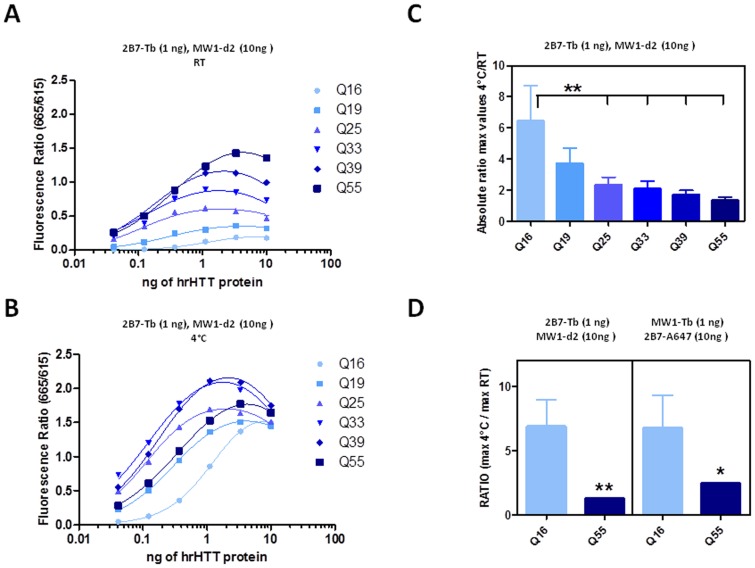
The temperature dependence of the 2B7-MW1 TR-FRET signal is inversely proportional to polyQ length. A.-B. Fluorescence signals obtained from dilution curves of N548 proteins bearing Q16, Q19, Q25, Q33, Q39 and Q55, tested by TR-FRET (1 ng/µl of 2B7-Tb and 10 ng/µl of MW1-d2) at RT (A) and 4°C (B). C. Ratio between the maximum value of the TR-FRET signal obtained at 4°C and the maximum value of the TR-FRET signal obtained at RT, referred to the curves presented in A and B. D. Ratio obtained and expressed as in C after performing TR-FRET with 2B7-Tb (1 ng/µl) and MW1-d2 (10 ng/µl) antibody combination (as before) or MW1-Tb (1 ng/µl) and 2B7-Alexa647 (10 ng/µl) antibody combination on N548 proteins bearing Q16 and Q55 repeats. In C and D data are represented as mean ± S.D. of three independent experiments; significance was calculated using the one-way ANOVA test and Bonferroni's multiple comparison post-test (** p<0.01 and * p<0.05).

**Figure 4:**
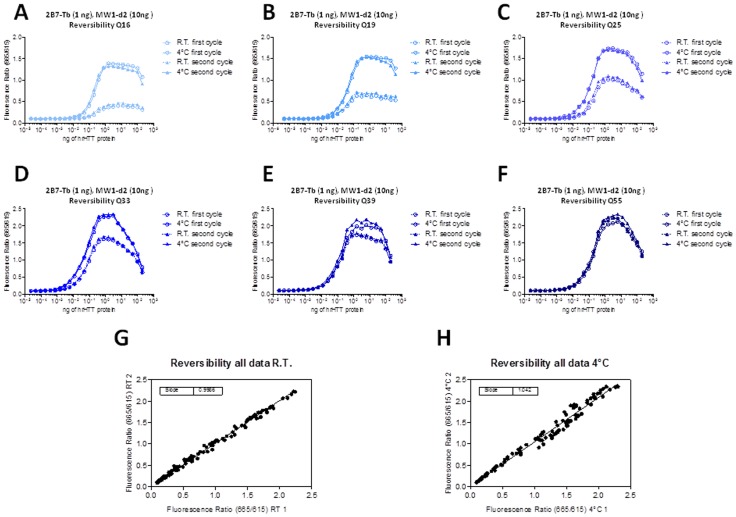
Reversibility of the temperature-dependent effect on TR-FRET signal. Following assembly of the TR-FRET cocktail (2B7-Tb and MW1-d2, 1∶10 ratio), the same samples were subjected to 2 successive cycles of incubation (for 1 hr) and measurement, at the two different temperatures (RT and 4°C). Note that the difference between fluorescence ratio values obtained at RT or 4°C is inversely correlated with polyQ length. A-F. Reversibility for HTT N548 HTT of increasing polyQ length (Q16, Q19, Q25, Q33, Q39, Q55). G. Correlation between fluorescence ratio values obtained in the two RT readings performed during the cycles. H. Correlation between fluorescence ratio values obtained in the two 4°C readings performed during the cycles.

We reasoned that the conformational flexibility of the N-terminal portion of HTT may be detected by TR-FRET assays by interrogating relevant epitopes. In the appropriate context, TR-FRET signals can inform on epitope accessibility as well as relative distance between the antibodies themselves ([28], [29]), and therefore can provide conformational information on the protein analyte. In the simplest context, we elected to investigate conformational flexibility in recombinant, purified HTT proteins by TR-FRET using an antibody pair comprising previously characterized monoclonals (2B7 and MW1; Fig. 1A; [13], [27], [30]–[32]) interrogating two regions of known relevance for HTT conformation and biological properties, namely the N17 domain and the polyQ domain (e.g. see [17], [24], [24], [26]). As one of the major factors influencing the stability of protein conformation is temperature (e.g. see[33]), we pragmatically chose this variable and decided to investigate HTT behaviour at a low temperature (4°C) and at the temperature at which TR-FRET assays are normally performed (room temperature, 20°C, RT). In order to avoid possible confounding effects due to aggregation, we chose to employ recombinant, purified N548 HTT fragments of different polyQ length, which should not aggregate or aggregate at a lower rate respective to the exon 1 under the conditions employed (Fig. 1B and C, [13]). Initially, we selected HTT proteins bearing two polyQ lengths, representing a wild type (Q16) and an expanded, or mutant (Q55), context. We then performed TR-FRET using antibodies 2B7 (labeled with a donor fluorophore, namely terbium) and MW1 (labeled with the acceptor fluorophore, D2), performing the incubation prior to reading at RT and then shifting the sample to 4°C for 2 hrs, first on a fixed (3.3 ng) and then on a range of HTT protein analyte concentrations (Fig. 2 A–E). As expected, the specific (donor-acceptor energy transfer dependent) fluorescence signal was significantly higher for mutant (Q55) HTT protein relative to wild-type (Q16) HTT protein (Fig. 2A). On this substrate, at RT the fluorescence signal obtained with the 2B7-Tb MW1-D2 antibody pair was close to that obtained from acceptor labeled MW1 alone (Fig. 2A). This is consistent with the specificity of the assay for mutant HTT protein ([13]). Interestingly, the TR-FRET signal obtained with 2B7-MW1 on wild type (Q16) N548 HTT protein can be significantly affected by temperature, where sample incubation at 4°C results in significantly higher TR-FRET signal than samples incubated at RT (Fig. 2B and C). However, when the samples containing the mutant (Q55) N548 HTT protein are incubated at 4°C the increase in TR-FRET signal was significantly reduced relative to the increase observed for the Q16 counterpart (Fig. 2B and C), suggesting that polyQ expansion influences the effects of temperature on protein conformation and/or antibody epitope recognition. The observed effect is not influenced by the stoichiometry of the donor and acceptor antibody pair, as performing the experiments with a 1∶1 instead of a 1∶10 ratio of donor to acceptor antibody pair, in which the acceptor-labeled MW1 is more limiting, still results in a temperature- and polyQ dependent variation in the obtained TR-FRET signal (Fig. 2D and E). A clear polyQ dependence of the temperature effect in the 2B7-MW1 TR-FRET signal was confirmed using HTT substrates of increasing polyQ length (Fig. 3A–C). Additionally, to further avoid the possibility of different MW1 binding sites between wild type and expanded HTT influencing the findings when using acceptor-labeled MW1, experiments were performed where 2B7 was labeled with acceptor label and MW1 with donor label. The results (Fig. 3D) indicate that the influence of temperature on TR-FRET signal using 2B7 and MW1 antibodies is independent of the nature of the acceptor antibody. The effect is rapidly (1 hr-cycles) reversible (Fig. 4), indicating its dependence on fast operating events compatible with conformational changes. We therefore concluded that, under these experimental conditions, temperature can influence HTT epitope exposure/recognition and/or protein analyte conformation, and investigated this further employing a non immunoassay-based orthogonal readout.

### HTT conformation is affected by temperature as measured by circular dichroism spectroscopy

**Figure 5:**
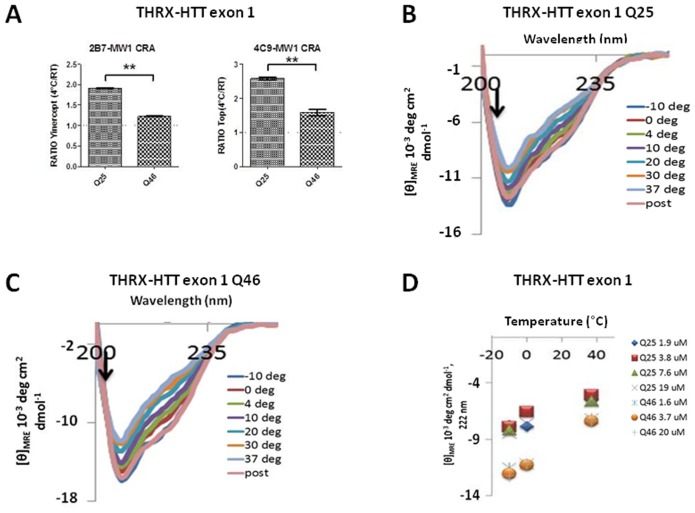
Temperature and polyQ length influence conformation of thioredoxin-exon 1 HTT proteins. A. Ratio between the maximum value of the TR-FRET curve obtained at 4°C and the maximum value of the TR-FRET curve obtained at RT, referred to the curves obtained with two different antibody combinations (2B7-MW1 and 4C9-MW1) on recombinant, purified thioredoxin-exon 1 HTT proteins employed for circular dichroism (CD) spectroscopy studies. CD spectra for THRX-HTT exon 1 Q25 (B) and Q46 (C) at all the temperatures tested showing an isosbestic point around 203 nm (black arrows) and showing the spectra for each construct at −10°C after measurements had been taken at 37°C to be nearly identical to those viewed beforehand, indicating the reversibility of the temperature effect. D. MRE values at 222 nm for THRX-HTT exon 1 Q25 and Q46 at indicated concentrations.

**Figure 6:**
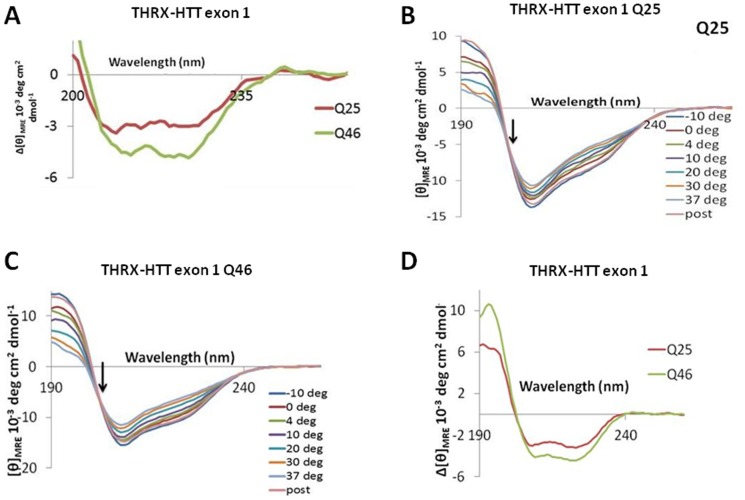
Temperature affects alpha-helical structure of thioredoxin-exon 1 HTT proteins. A. Difference spectra between −10°C and 37°C, the temperature extremes shown in parts B and C of Figure 5, for Q25 and Q46. CD spectra plotted using MRE from 190 to 260 nm for THRX-HTT exon 1 Q25 (B) and Q46 (C) in salt-free phosphate buffer at all the temperatures tested showing an isosbestic point around 203 nm (black arrows) and reversibility of structural changes upon cooling from 37°C to −10°C (post). D. Difference spectra between −10°C and 37°C for Q25 and Q46 tested in parts B and C. Each of these spectra shows a maximum near 190 nm and minima at 208 and 222 nm.

**Figure 7:**
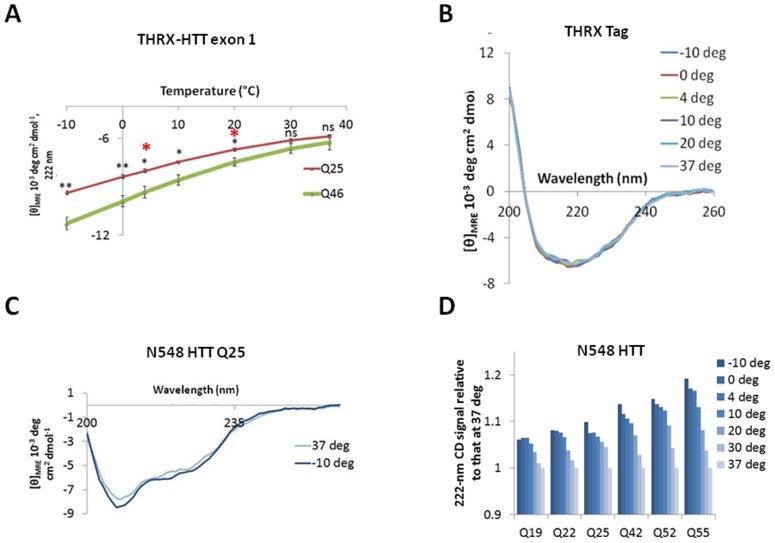
Temperature-dependent alpha-helical variations in thioredoxin-tagged exon 1 HTT proteins are not due to variations in tag helicity, and are also observed in untagged, larger N548 HTT purified recombinant proteins. A. MRE values at 222 nm (at which alpha-helical structure has a signature peak) for Q25 and Q46 THRX-HTT exon 1 at each temperature tested. Red asterisks denote measurements taken at 4°C and RT, the temperatures used in the TR-FRET experiments. Black asterisks denote *p*-values as determined by the Student's *t*-test. *p<0.05. **p<0.01. ns, not significant. All data are represented as mean ± S.D. of three independent experiments. B. CD spectra for the THRX fusion partner alone at indicated temperatures. C. MRE spectra for the Q25 N548 HTT protein at −10°C and 37°C, showing that the temperature effect can still be seen in this longer fragment. D. MRE values at 222 nm at each indicated temperature relative to MRE at 37°C for N548 HTT of increasing polyQ length (Q19, Q22, Q25, Q42, Q52, Q55).

Circular dichroism spectroscopy (CD) measures differences in absorption of left-handed polarized light versus right-handed polarized light due to structural asymmetry in the analyte, and can inform on the presence of secondary/tertiary structure in proteins (e.g., see [18]). CD data report on the overall secondary structure content with contributions from all amino acids in the protein sequence. As the spectral contribution of residues around the polyQ region is progressively diminished with increasing HTT fragment length, we initially decided to investigate temperature effects on HTT proteins of different polyQ lengths using the shorter exon 1 fragments. These fragments were produced recombinantly in bacteria as previously described [18]. These proteins, available with normal (Q25) or expanded (Q46) polyQ regions, bear a thioredoxin N-terminal fusion (THRX-HTT) to improve stability and solubility. We first sought to determine whether such a heterologous N-terminal tag fused to the shorter HTT exon 1 fragment might affect the temperature- and polyQ-dependent variation in 2B7-MW1 TR-FRET values. The results, employing the 2B7-MW1 antibody pair and confirmed with a second antibody pair (4C9-MW1), indicated that a temperature-dependent, polyQ-dependent variation in TR-FRET signal is still observable with THRX-HTT exon 1 Q25 protein, while it is virtually absent when THRX-HTT exon 1 Q46 protein is used (Fig. 5A), coherent with what was observed using the longer N548 HTT proteins as substrate. We therefore proceeded with investigating the effects of temperature on the structure of THRX-HTT exon 1 proteins bearing 25 or 46 Q and chose to perform the experiments at 37°C and −10°C in order to explore a wide temperature range that encompassed the conditions employed in the TR-FRET assays. The ensuing CD spectra for Q25 and Q46 have significant changes with temperature that are reversible (Fig. 5B and C) and independent of protein concentration in the range used (Fig. 5D), consistent with the results of the immunoassay-based conformational assay. The difference spectra for both proteins exhibit characteristic minima at 208 and 222 nm indicating increased formation of alpha-helical structure at lower temperatures, which is more pronounced for Q46 HTT protein (Fig. 6A), and all spectra (Fig. 5B and C) show an isosbestic point near 203 nm, which is commonly observed for conversions between random coil and alpha-helical structure. Another key feature of alpha-helical CD spectra is a maximum near 190 nm, which was not possible to view at high salt concentrations. We therefore recorded CD spectra for both constructs in salt-free phosphate buffer. These spectra (Fig. 6B and C) also show isosbestic points near 203 nm, and the difference spectra show the peaks near 190, 208, and 222 nm that are indicative of an alpha-helix (Fig. 6D). The temperature dependent change in alpha-helicity is frequently plotted using the mean residue ellipticity (MRE) observed at 222 nm. Using this measure, we find that cooling increases helicity more strongly for the Q46 than the Q25 derivative (Fig. 7A; red asterisks indicate measurements performed at 4°C and RT, for direct comparison with TR-FRET data). The THRX tag is highly stable requiring more than 4 M urea for denaturation [34]. In agreement with this notion, control experiments with the fusion tag alone revealed no detectable change in the CD spectra between −10 and 37°C (Fig. 7B), indicating that the exon-1 portion of the fusion protein contains the region that undergoes structural changes with temperature. The consistent isosbestic point across temperatures (Fig. 5B and C and 6B and C) together with the typically alpha-helical difference spectra (Fig. 6A and D) suggest that the conformational changes are dominated by a helix-coil transition. We therefore applied a previously developed helix-coil model as one means to calculate the number of residues that experienced a change in helicity [35], [36]. Using this model, we estimate that on the order of 23 and 31 residues become helical upon cooling for the Q25 and Q46 constructs, respectively (Table 1A). To further confirm the differential conformational changes in these constructs, we analyzed their CD spectra using DICHROWEB software [37], [38] and thereby again found the Q46 protein to experience a greater change in helicity, with estimates of 21 to 23 residues and 30 to 33 residues changing in Q25 and Q46, respectively (Table 1A). From DICHROWEB analysis, both constructs are also seen to undergo a loss in beta-sheet upon cooling (Table 2); however, this change is relatively small and cannot readily be identified in the difference spectra. All alpha-helicity estimates for the HTT Q46 protein are far beyond the number of amino acids that are contained in the N17 domain, a region which has a propensity to adopt an alpha-helical structure [24] ([39], [40]). This suggests that the observed change in helicity cannot exclusively stem from structural changes in the N17 region and likely involves the polyQ region (see below). The PRR can adopt a polyproline type II (PPII) structure (e.g. [24] [19], [41]), a conformation whose stability may also increase with decreasing temperature (e.g. see [18], [33]). However, the CD spectrum of a PPII structure is entirely different from that of an alpha-helix. While we cannot exclude that more subtle changes might have also occurred in the PRR, the structural changes observed by CD are dominated by alpha-helical structure (Fig. 6A and D). Although the temperature-dependent change in helicity is more pronounced for Q46, it is important to note that the overall helicity still remains higher at 37°C. Thus, Q46 has a higher helicity at all temperatures and this enhanced structural ordering is likely related to the apparent enhanced rigidity observed by the CRA on polyQ expanded HTT proteins. As TR-FRET observations employed N548 proteins, we next sought to determine if such HTT protein fragments would display CD profiles compatible with observations made using THRX-HTT exon 1 fragments. Although the impact of the polyQ region on the CD spectra is significantly diluted out in the larger N548 construct, we were, nonetheless, able to determine temperature-dependent changes by CD on N548 Q25 HTT protein (Fig. 7C). Again, we found that MRE values became more negative with decreasing temperature, indicating increased alpha-helical structure at lower temperatures. Coherently with the immunoassay-based observations, an influence of polyQ length on alpha-helical content of N548 HTT proteins was also observed (Fig. 7D). Collectively, these data pertaining to exon-1 and N548, two constructs of very different lengths, are compatible with a role for both temperature and polyQ expansion on huntingtin conformation, confirming the observation obtained using immunoassay-based approaches.

**Table 1:** Number of amino acids that gain alpha-helicity upon cooling from 37°C to −10°C, using different algorithms.

	*Helix-Coil Model*	*SELCON3*	*CONTIN*
**Q25**	23	21	23
**Q46**	31	33	30

Estimation of temperature-dependent structure of thioredoxin exon 1 HTT proteins. A. Number of amino acids in HTT exon 1 of each Q-length that experienced a temperature-dependent change in alpha-helical structure, as indicated, calculated either by the helix-coil model or using DICHROWEB software. Taking the varying results together, it is estimated that between 21 and 27 amino acids in the Q25 construct and between 30 and 37 amino acids in the Q46 construct gain alpha-helical structure upon cooling from 37°C to –10°C.

**Table 2:** Percentage (and number) of amino acids in a given conformation at indicated temperature obtained from SELCON3 and CONTIN algorithms.

	*Conformation*	*−10°C, SELCON3*	*37°C, SELCON3*	*−10°C, CONTIN*	*37°C, CONTIN*
**Q25**	alpha-helix	32 (74)	23 (53)	33 (76)	23 (53)
	beta-sheet	16 (37)	20 (46)	17 (39)	22 (51)
	turns	15 (35)	15 (35)	13 (30)	14 (32)
	unordered	37 (85)	39 (90)	37 (85)	41 (94)
**Q46**	alpha-helix	37 (93)	24 (60)	38 (95)	26 (65)
	beta-sheet	12 (30)	20 (50)	15 (38)	21 (53)
	turns	15 (38)	15 (38)	13 (33)	14 (35)
	unordered	34 (85)	39 (98)	35 (88)	40 (100)

Estimated secondary structure percentages of amino acids in each construct at indicated temperatures according to DICHROWEB analyses. The corresponding number of amino acids is given in parentheses. While the temperature-dependent changes are predominantly alpha-helical, small changes in beta-sheet structure are also estimated. The number of residues that adopt a turn structure remains virtually unchanged. Note that the fusion proteins contain a Trx tag, which based upon the Trx crystal structure (PDB: 1F6M) has 39 and 31 amino acids that adopt an alpha-helical and a beta-sheet structure, respectively [70]. Thus, much of the alpha-helical and beta-sheet structure detected in the fusion proteins by DICHROWEB is contributed by the Trx moiety.

### Role of the polyQ domain in temperature-dependent conformational changes in HTT proteins detected by TR-FRET

**Figure 8:**
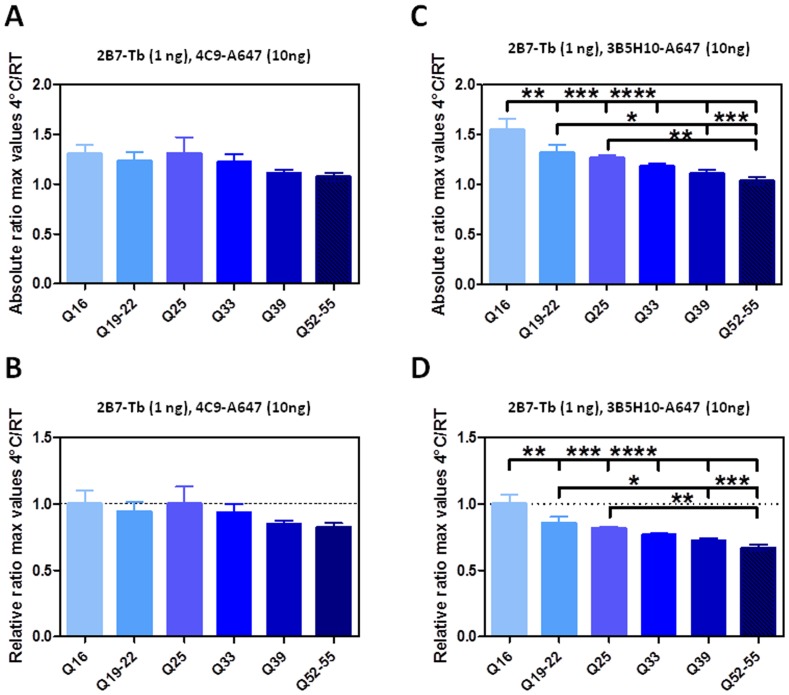
Role of the polyQ domain in temperature-dependent conformational changes in hrHTT proteins detected by TR-FRET. A. Absolute ratios between the maximum value of the TR-FRET signal obtained at 4°C and the maximum value of the TR-FRET signal obtained at RT, referred to the curves obtained with an antibody combination which does not interrogate directly the polyQ region (2B7 Tb, 4C9-A647, 1∶10 ratio) on a dilution curve of hrN548 Q16 and Q55 proteins. B. Relative ratios, normalized with respect to ratios obtained for Q16 protein, for the same samples, illustrating the absence of a temperature- and polyQ-dependent effect obtained with this antibody combination. C. Absolute ratios between the maximum value of the TR-FRET signal obtained at 4°C and the maximum value of the TR-FRET signal obtained at RT, referred to the curves obtained with 2B7-Tb, 3B5H10-A647 (1∶10 ratio) on hrN548 Q16 and Q55 proteins. D. Relative ratios, normalized with respect to ratios obtained for Q16 protein, for the same samples, illustrating the presence of a temperature- and polyQ-dependent effect with this antibody combination. All data are represented as mean ± S.D. of three independent experiments, significance was calculated using the one-way ANOVA test and Bonferroni's multiple comparison post-test (* = p<0.05; ** = p<0.01; *** = p<0.005; **** = p<0.001).

Based on the CD spectroscopy data, we reasoned that the most significant conformational change conferred on HTT by temperature variations and detected by TR-FRET would affect the N-terminal portion of the protein, and would be the strongest when interrogating polyQ epitopes. Therefore, we investigated whether an interrogation of two epitopes straddling (but not within) the polyQ region would result in detection of a temperature-dependent conformational effect, which as we described above is strongest for unexpanded polyQ (wild type) HTT. The 2B7 and 4C9 epitopes are specific for epitopes within the N17 and PRR regions of HTT, respectively [13], [32]. Using this antibody pair, we investigated the effects of temperature on TR-FRET signals obtained on N548 recombinant, purified proteins with Q16 and Q55 lengths, as performed above for the 2B7 and MW1 antibody pair. The 2B7-4C9 antibody pair does not detect a significant temperature-dependent conformational effect on HTT protein (Fig. 8A and B), lending support to the notion that the conformational change (alpha-helical content change) observed by CD spectroscopy is principally affecting the polyQ domain, with little detectable effect on other HTT regions. Consistent with this notion, employing 2B7 in combination with 3B5H10, another antibody specific for a polyQ epitope (e.g. [31], [42]) resulted in detection of the temperature-dependent, polyQ-dependent variation in TR-FRET signals on the same protein analytes (Fig. 8C and D). We further extended this analysis to determine whether recognition of the N17 domain (with 2B7) is a critical factor in the conformational change detected in TR-FRET with 2B7-MW1 using a variety of antibody pair combinations (including the use of 4C9 as a donor). The results clearly indicate that detection of the conformational variation is critically dependent on interrogation of the polyQ region (Table 3).

**Table 3:** TR-FRET-based detection of conformational rigidity in HTT proteins can be detected when one of the antibodies in the TR-FRET pair detects the polyQ region.

*Donor antibody*	*Acceptor antibody*	*Epitopes*	*Temperature and polyQ-dependent TR-FRET variation*
**2B7**	MW1	N17, polyQ	+
**MW1**	2B7	polyQ, N17	+
**2B7**	2166	N17, ∼aa 445-459	−
**2B7**	4C9	N17, polyP	−
**2B7**	3B5H10	N17, polyQ	+
**4C9**	MW1	polyP, polyQ	+
**4C9**	2166	PolyP, ∼aa 445-459	−

Donor (Tb) and acceptor (d2 or Alexa647) labeled antibodies are indicated, as well as the epitopes recognized. Further details of antibody characterization and references thereof can be found in the materials and methods section.

### Conformational effects measured by the 2B7-MW1 TR-FRET assay can be observed in HTT expressed in mammalian cells, and is independent of HTT fragment length

**Figure 9:**
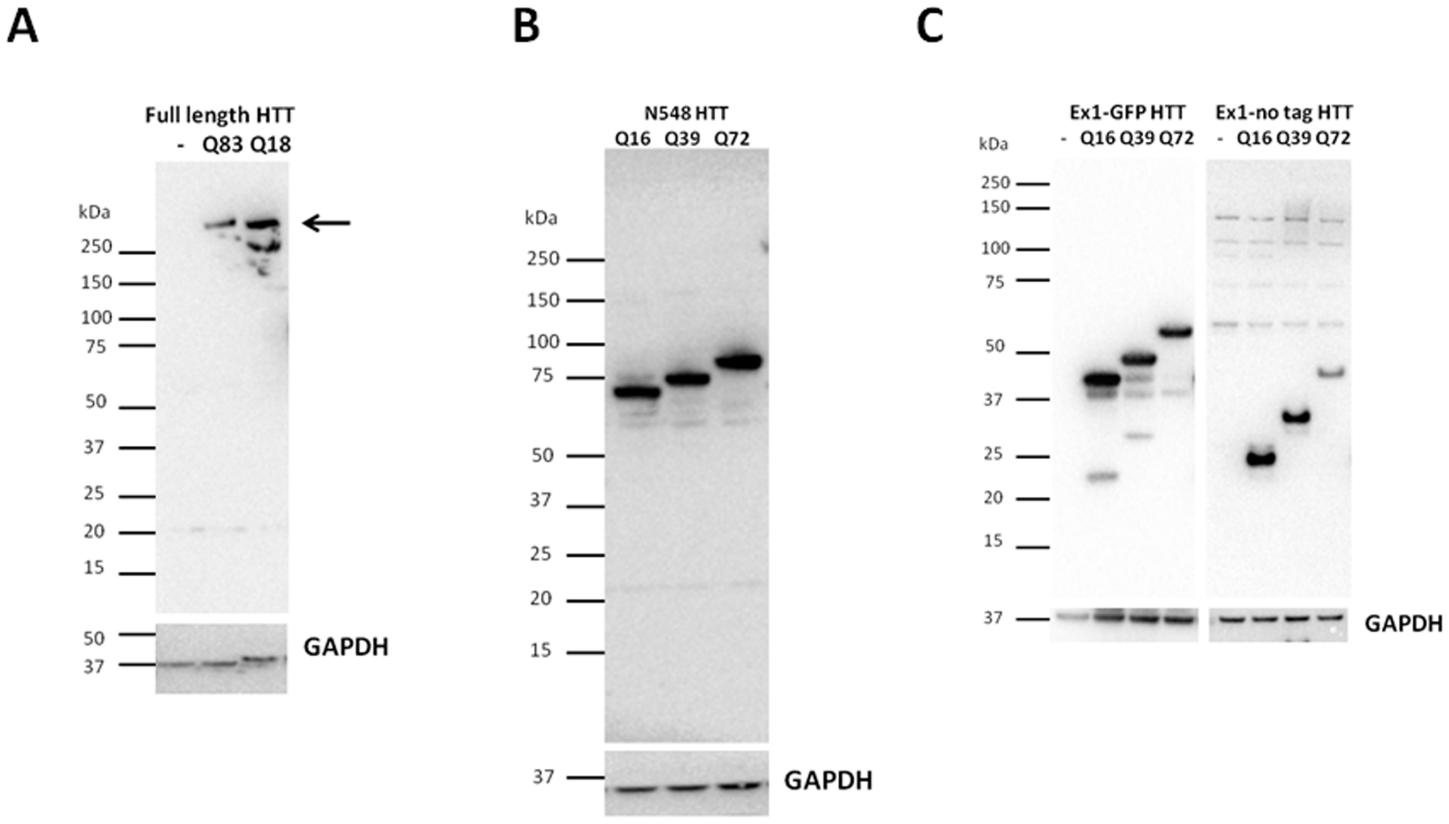
HTT proteins of defined fragment length and polyQ content expressed in transfected cells. Western blot of lysates from transfected HEK293T cell samples probed with antibody 4C9, demonstrating expression of constructs encoding full length HTT with Q18 or Q83 (arrow), N548 HTT and exon-1 HTT with or without C-terminal EGFP with Q16, Q39 and Q72. Blots were also probed with an anti-GAPDH antibody as loading control.

**Figure 10:**
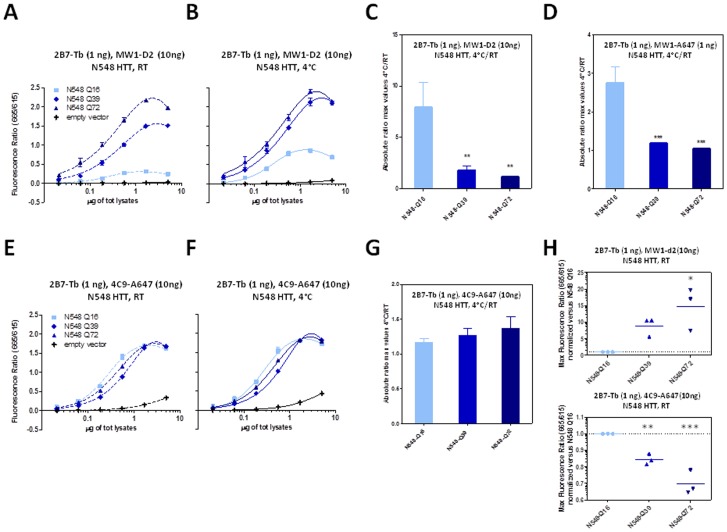
Temperature- and polyQ- dependent conformational changes detected on HTT N548 protein (Q16, Q39 and Q72) using the 2B7-MW1 TR-FRET assay in a biological sample (lysates from transfected cells). A–B. TR-FRET values obtained at RT (A) or 4°C (B) using 2B7-Tb (1 ng/µl) - MW1-d2 (10 ng/µl) on different protein concentrations of lysates, confirming the temperature- and polyQ-dependent conformational effect. C. Absolute ratios between the maximum value of the TR-FRET signal obtained at 4°C and the maximum value of the TR-FRET signal obtained at RT, referred to the curves in A and B. D. Absolute ratios between the maximum value of the TR-FRET signal obtained at 4°C and the maximum value of the TR-FRET signal obtained at RT, referred to the curves achieved using a 1∶1 ratio of 2B7-Tb and MW1-d2 (1 ng/µl for both the antibodies). E-F. TR-FRET values obtained at RT (E) or 4°C (F) using 2B7-Tb and 4C9-Alexa647 on the same lysates, confirming the requirement for polyQ interrogation to observe conformational changes. G. Absolute ratios between the maximum value of the TR-FRET signal obtained at 4°C and the maximum value of the TR-FRET signal obtained at RT, referred to the curves in G and F. H. Maximum value of the TR-FRET signals obtained at RT, normalized with respect to the maximum value of the TR-FRET signals obtained for N548 Q16, referred to the curves in A (upper graph) and E (lower graph). In C, D and G, data are represented as mean ± S.D. of three independent experiments. In H the three different experiments are represented as single point together with their mean. Significance was calculated using the one-way ANOVA test and Bonferroni's multiple comparison post-test (* = p<0.05; ** = p<0.01; *** = p<0.005).

**Figure 11:**
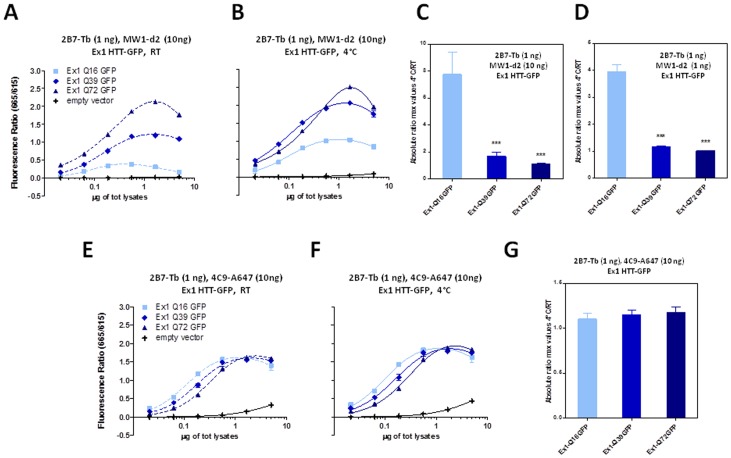
Temperature- and polyQ- dependent conformational changes detected on HTT exon 1 protein (Q16, Q39 and Q72) using the 2B7-MW1 TR-FRET assay in a biological sample (lysates from transfected cells). A–B. TR-FRET values obtained at RT (A) or 4°C (B) using 2B7-Tb (1 ng/µl) - MW1-d2 (10 ng/µl) on lysates, confirming the temperature- and polyQ-dependent conformational effect. C. Absolute ratios between the maximum value of the TR-FRET signal obtained at 4°C and the maximum value of the TR-FRET signal obtained at RT, referred to the curves in A and B. D. Absolute ratios between the maximum value of the TR-FRET signal obtained at 4°C and the maximum value of the TR-FRET signal obtained at RT, referred to the curves achieved using a 1∶1 ratio of 2B7-Tb and MW1-d2 (1 n/µl for both the antibodies). E-F. TR-FRET values obtained at RT (E) or 4°C (F) using 2B7-Tb and 4C9-Alexa647 on the same lysates, confirming the requirement for polyQ detection to observe conformational changes. In C, D and G data are represented as mean ± S.D. of three independent experiments; significance was calculated using the one-way ANOVA test and Bonferroni's multiple comparison post-test (** = p<0.01; *** = p<0.005).

**Figure 12:**
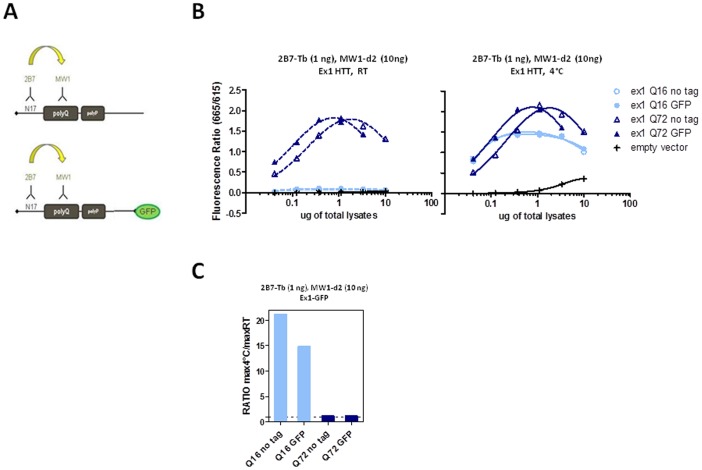
Presence of a C-terminal heterologous tag does not influence the temperature- and polyQ dependent conformational effect. A. Scheme of proteins expressed by constructs encoding exon 1 HTT cDNAs with or without C-terminally fused EGFP. B. TR-FRET values (665 nm/615 nm) obtained at RT or 4°C using 2B7-Tb (1 ng/µl)- MW1-d2 (10 ng/µl) on lysates of HEK293T cells transfected with exon 1 HTT expression constructs (Q16, Q39 or Q72) with or without a C-terminal EGFP moiety, confirming the temperature- and polyQ-dependent conformational effect irrespective of the presence of a C-terminal EGFP fusion. C. Absolute ratios between the maximum value of the TR-FRET signal obtained at 4°C and the maximum value of the TR-FRET signal obtained at RT, referred to the curves in C.

**Figure 13:**
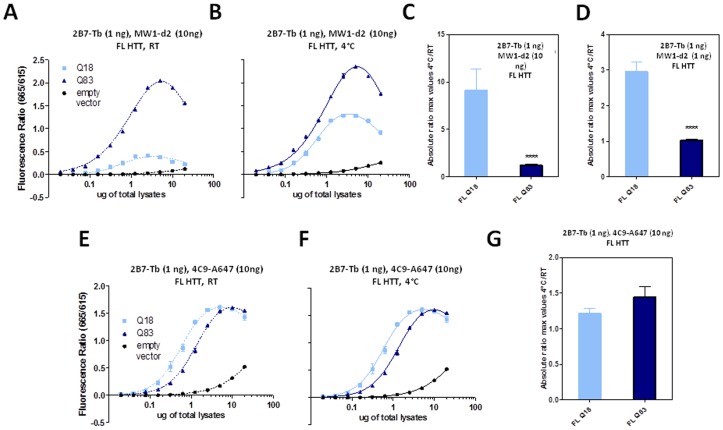
Temperature- and polyQ- dependent conformational changes detected on HTT full length protein (Q18 and Q83) using the 2B7-MW1 TR-FRET assay in a biological sample (lysates from transfected cells). A–B. TR-FRET values obtained at RT (A) or 4°C (B) using 2B7-Tb (1 n/µl) - MW1-d2 (10 ng/µl) on lysates, confirming the temperature- and polyQ-dependent conformational effect. C. Absolute ratios between the maximum value of the TR-FRET signal obtained at 4°C and the maximum value of the TR-FRET signal obtained at RT, referred to the curves in A and B. D. Absolute ratios between the maximum value of the TR-FRET signal obtained at 4°C and the maximum value of the TR-FRET signal obtained at RT, referred to the curves achieved using a 1∶1 ratio of 2B7-Tb and MW1-d2 (1 n/µl for both antibodies). E-F. TR-FRET values obtained at RT (E) or 4°C (F) using 2B7-Tb and 4C9-Alexa647 on the same lysates, confirming the requirement for polyQ detection to observe conformational changes. In C, D and G data are represented as mean ± S.D. of three independent experiments; significance was calculated using the one-way ANOVA test and Bonferroni's multiple comparison post-test (** = p<0.01; *** = p<0.005).

Having investigated the temperature- and polyQ-dependent conformational change using recombinant, purified HTT proteins of different lengths (N548 and exon 1), we investigated the effect in HTT proteins expressed in a biological context, using lysates from HEK293T cells transiently expressing wild type or mutant HTT cDNAs of different lengths (full length, N548 or exon 1; Fig. 9A, B and C, respectively). In order to demonstrate temperature- and polyQ-dependence of the effect in HTT proteins expressed in a cellular context (rather than on purified recombinant proteins), we elected to use a transient transfection system which, although clearly artificial, allows rapid and robust expression of HTT proteins of defined length and polyQ content, as produced by selected and characterized plasmid constructs. We analyzed these lysates using the same 2B7-MW1 antibody pair used on recombinant proteins (see above), as well as a control antibody pair that did not detect the temperature- and polyQ-dependent conformational change (2B7-4C9). Consistent with our results using recombinant, purified N548 HTT proteins, the conformational change was observed when lysates from HEK293T cells transfected with an HTT construct encoding the N548 fragment were analyzed using 2B7-MW1 (Fig. 10A-D), but not when the same lysates were analyzed with the 2B7-4C9 antibody pair (Fig. 10E–G). As expected, the difference in behaviour between the MW1 and 4C9 antibodies on wild type and mutant HTT substrates was clearly observed, with the acceptor-labeled MW1 producing an increased TR-FRET signal with increasing polyQ length and the acceptor-labeled 4C9 displaying a decreased TR-FRET signal with increasing polyQ length (Fig. 10H), consistent with published observations [26]. Similar results were obtained with lysates from HEK293T cells transfected with a construct encoding exon 1 HTT fragments (Fig. 11). As the employed exon 1 HTT constructs included a carboxyl-terminal (C-terminal) EGFP fusion, we investigated the effects of the presence of a heterologous protein domain (EGFP) on detection of the temperature- and polyQ-dependent conformational change by comparing EGFP tagged and untagged exon 1 HTT expression constructs, transiently transfected in HEK293T cells. The results (Fig. 12) clearly suggested that the detected conformational change was again independent of regions outside the N-terminal part of HTT, even when such regions are heterologous. Finally, we investigated the conformational effect in cell lysates expressing full length (Q18, Q83) HTT. Again, the temperature- and polyQ-dependent conformational change was observed when lysates from HEK293T cells transfected with an HTT construct encoding the full length protein were analyzed using 2B7-MW1, but not when the same lysates were analyzed with the 2B7-4C9 antibody pair (Fig. 13). When considered with the data demonstrating that N-terminal fusion with thioredoxin does not prevent detection of the conformational change, the data collectively suggest a pronounced conformational flexibility of the N-terminal portion of HTT that is not affected by natural or heterologous flanking sequences.

### N17 mutations do not significantly influence temperature-dependent conformational changes in HTT

**Figure 14:**
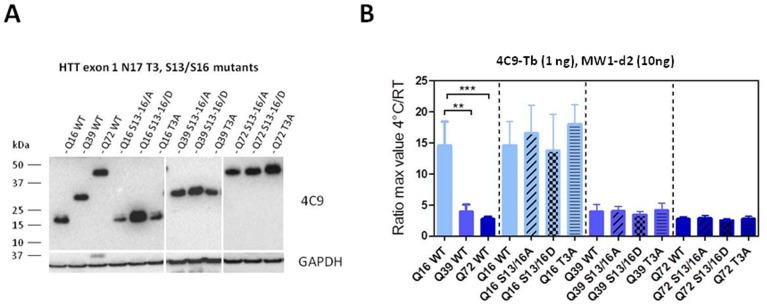
N17 mutations do not affect temperature- and polyQ- dependent conformational changes as detected by a 4C9-MW1 antibody combination in TR-FRET. A. Western blot of lysates from transfected HEK293T cell samples probed with antibody 4C9, demonstrating expression of constructs encoding wild type or N-terminal mutant HTT exon 1 overexpressed in HEK293T cells with Q16, Q39 and Q72. The blot was also probed with an anti-GAPDH antibody as loading control. B. Absolute ratios between the maximum value of the TR-FRET signal obtained at 4°C and the maximum value of the TR-FRET signal obtained at RT, referred to the curves obtained using 2B7-Tb (1 ng/µl) and MW1-d2 (10 ng/µl) on the same lysates. Data are represented as mean ± S.D. of three independent experiments; significance was calculated using the one-way ANOVA test and Bonferroni's multiple comparison post-test (** = p<0.01; *** = p<0.005).

We next asked if variations within the N-terminal portion of HTT, and specifically within the N17 domain, could influence the temperature- and polyQ-dependent conformational event detected by the 2B7 and MW1 TR-FRET antibody combination. Post-translational modifications represent an obvious candidate modifier of mutant HTT properties, in particular those addressing the first 17 amino acids of the protein, as this region has been shown to have profound influences on subcellular localization, aggregation and toxicity (e.g. reviewed in [43], [44]). Mutations within the N17 region have been utilized to mimic the presence of PTMs; for instance, D mutations of T3, S13 and S16 residues mimic phosphorylation at these positions and have been used to provide support towards the hypothesis that N17 phosphorylation can influence HTT properties pertinent to toxicity of the mutant protein [17], [20], [23], [26], [45]–[47]. In order to determine if mutational phosphomimicry can affect HTT conformation as detected by the temperature-dependent TR-FRET assay, we employed lysates from HEK293T cells transfected with HTT exon 1 cDNA constructs bearing T3, S13 and S16 mutations in a Q16, Q39 and Q72 context (Fig. 14A). Instead of employing the 2B7-Tb/MW1-D2 antibody pair, we employed a 4C9-Tb-MW1-D2 antibody pair in order to avoid potential confounding issues arising from the use of 2B7, whose epitope is within the N17 region (where the residues affected by the mutations are present). When analyzed with the 4C9-MW1 TR-FRET assay, none of the mutants influenced the temperature-dependent behaviour of either wild type (Q16) or mutant (Q39 and Q72) HTT exon 1 (Fig. 14B). Together with the observation that the detected conformational event is independent of HTT fragment length (see Figs. 10, 11, 12, and 13), this finding suggests that it may also be independent of changes within the N17 region.

## Discussion

**Figure 15:**
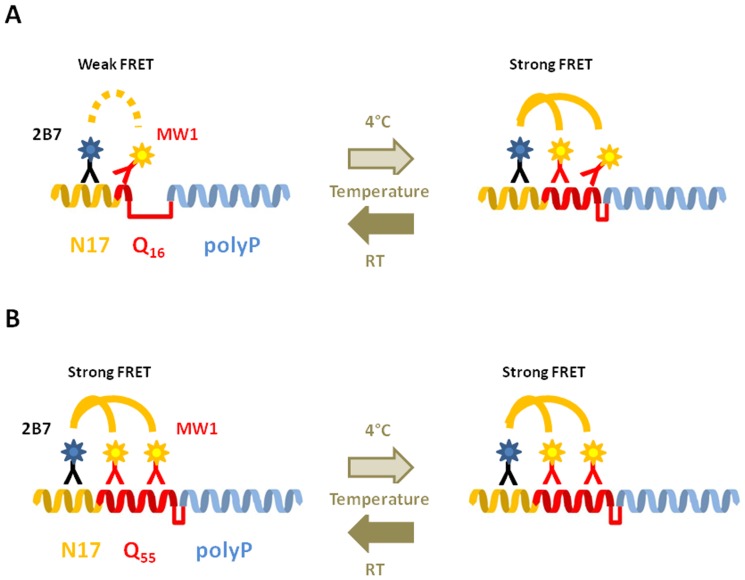
Model of structural changes observed in HTT proteins upon temperature change. The N17 (orange) and polyproline (blue) regions are depicted as alpha- and PPII helices, respectively, while the polyglutamine region is illustrated as a domain which can adopt temperature- and polyQ-dependent conformations (red). The epitopes of the 2B7 and MW1 antibodies are graphically illustrated. A. The N-terminal region of a HTT protein bearing a wild type (e.g. Q16) polyQ expansion can adopt variable degrees of structure in the polyQ region, with alpha-helical content being higher at lower temperatures. At higher temperatures, alpha-helical content is lower and results in a 1: ≤1 ratio of 2B7Tb-MW1D2 bound to HTT. At lower temperatures, this ratio becomes 1: ≥1 due to increased alpha-helical content in the polyglutamine region, an increased number of epitopes for the acceptor-labeled MW1 antibody and, consequently, an increase in TR-FRET signal. B. In the N-terminal region of a HTT protein bearing a mutant (e.g. Q55) polyQ expansion, the polyQ region adopts a higher alpha-helical content relatively to wild type HTT, a relatively larger number of epitopes for the acceptor-labeled MW1 antibody are present and, consequently, TR-FRET signals are higher. The significant alpha-helical structure of the mutant polyQ region is less influenced by temperature, the number of MW1 epitopes remains relatively constant and therefore FRET between donor and acceptor labels is relatively unchanged.

The N-terminal portion (exon 1 encoded) of the HTT protein is a key determinant of HD as it comprises the polyQ repeat expansion. Structural, biophysical, and functional studies of HTT exon 1 suggest that in the HTT monomer this region can be viewed as a modular unit comprised of at least three major structural domains — the N-terminal N17, the polyQ, and the PRR [24] [17]–[19], [25], [41], [48]. The N17 region displays a propensity to adopt a right handed amphipathic alpha-helical structure [24] [24], [39], [40]. This region is followed by the polyQ repeat module, which can take up a beta-sheet structure in fibrils, but which has been thought to be predominantly unfolded in solution (reviewed in [25], [49]). The polyQ module is immediately followed by a pure proline repeat encoding region which is likely to adopt a left-handed polyproline II helix structure [19], [41]. In both crystallographic and computational structural studies, the less ordered polyQ repeat is conformationally influenced by the flanking regions (which can form, interestingly, two protein helices of opposite handedness), with its N-terminal residues amenable to adopting the right-handed alpha-helical structure of the N17 module while its C-terminal residues can adopt the structure of the proline repeat module[24] [25], [48]. The N17 and PRR domains can have a substantial (and opposing) effect on the intramolecular and intermolecular properties of N-terminal HTT, with consequences on protein aggregation and cell toxicity [26], [50]–[55]. This is likely to result at least in part from structural interplay amongst the three modules, allowing a range of possible conformations and intramolecular/intermolecular interactions for N-terminal HTT fragments [55], [56]. Indeed, recent evidence proposes that the N-terminal portion of monomeric, wild type HTT is conformationally flexible, with the relatively unstructured polyQ domain acting as a flexible hinge between the N17 and downstream HTT domains (e.g. the PRR region), and becomes rigid when expanded in mHTT [26], [57]. This conformational dynamism is likely essential to modulate a range of intra- and intermolecular interactions which, when abnormal, might result in altered protein associations and abnormal HTT function [26]. The possibility of interrogating HTT conformation with simple robust assays would allow investigations on the role of mHTT conformational changes in HD, its determinants (e.g. post-translational modifications, protein-protein interactions) and its modulation (genetic or pharmacological) to influence mutant protein function, aggregation, and/or toxicity. With appropriate antibodies, TR-FRET assays provide the sensitivity, robustness and scalability required for screening applications. We have previously developed TR-FRET assays to quantify different HTT conformers [13] and reasoned that such assays might be able to detect conformational changes in response to variations in polyQ length and/or conditions known to modulate protein conformation, such as temperature. We reasoned that interrogating HTT epitopes including the polyQ repeat region would provide the most significant information, as this latter represents the pathologically relevant module. Using such immunoassays, we identified a reversible, polyQ-dependent effect of temperature on the TR-FRET signal of recombinant, N548 HTT proteins bearing wild-type or mutant polyQ expansions. Lowering the incubation temperature increased TR-FRET signal when wild type HTT was employed, and this increase was inversely proportional to polyQ length, reaching a minimum in proximity of the CAG length associated with HD (e.g. see [58], [59]). The rapid reversibility of the effect is compatible with a conformational event, and suggests decreased flexibility of mutant HTT relative to the wild type protein, consistent with recent observations [26]. Interestingly, the effect appears to be detectable only when the polyQ domain is interrogated, and is not influenced by HTT fragment length or by mutations affecting neighbouring domains (such as the N17 domain). Importantly, levels of soluble antigen are not significantly different at 4°C vs room temperature, as detected by TR-FRET interrogating epitopes other than the polyQ region. There are several possible explanations for the observed temperature- and polyQ-dependent differences in TR-FRET signals. These can be broadly grouped into effects impacting the antibodies used in the assay and effects impacting the antigen (HTT). As for the first, we cannot a priori exclude that the observed TR-FRET results are due to temperature-dependent changes in the affinity, flexibility, binding modality, position and number of labels and/or conformation of the IgGs, although such effects would have to be exquisitely specific for MW1 and 3B5H10 as the temperature- and polyQ-dependent TR-FRET effect is not observed when IgGs other than MW1 and 3B5H10 are used, even if labeled and employed in the same exact way (e.g. 2B7, 4C9 and 2166). A similar consideration applies to potential effects due to influences due to IgG conjugation aspects. However, the temperature-dependent kinetics of antibody-antigen interactions are indeed variable from antibody to antibody, and these kinetics are likely to be complex with antibodies such as MW1 which can recognize multiple epitopes in polyQ regions. For example, the affinity or avidity of MW1 for its epitope may be higher at lower temperature (for instance, through more stable hydrogen bonds) and such difference might be more readily detectable by TR-FRET on polyQ substrates where fewer epitopes are present and the signal is lower (e.g. wild type HTT). On substrates with longer polyQ regions, where multiple MW1 binding events are possible, this antibody-specific effect of temperature might be overridden by multiple IgGs binding to the (mutant) HTT antigen. A detailed investigation of the effects of temperature on the affinity and avidity of MW1 and 3B5H10 would ideally need to be performed in solution to be comparable to the TR-FRET assay, and under conditions where HTT conformation would need to be irreversibly fixed in order to eliminate antigen conformation as a confounding variable. The complexities of such analysis led us to apply an orthogonal, non-immunoassay biophysical readout to determine if temperature could influence antigen (HTT) conformation in a polyQ-dependent fashion. Our CD studies indicate that HTT exon 1 can take up an extensive helical structure that likely includes the N17 as well as substantial portions of the polyQ region. This notion is in agreement with prior crystallographic studies, which showed that alpha-helical structure could extend from the N17 into the polyQ region in some, but not all structures [24] [25 [24]]. Our data indicate that the observed alpha-helical and unfolded structures are in equilibrium with one another. Perhaps not surprisingly, lower temperatures favor the helical state. This effect is more pronounced at higher Q length and, additionally, higher Q lengths result in overall higher helicity at any given temperature. One possibility is that this residual helical structure directly causes apparent rigidity in proteins with higher Q lengths, a notion consistent with previous fluorescence studies which showed that intramolecular FRET is strongly polyQ length dependent [26 [26]]. However, we cannot exclude other more indirect mechanisms. For example, it is possible that epitope recognition by MW1 (and 3B5H10) is somewhat influenced by the extent of alpha-helical structure present in the polyQ region (Fig. 15). For instance, polyQ alpha-helical content could influence the exposure, number and/or relative distance/orientation of epitopes from the donor (Tb labeled) antibody. We are therefore further investigating the observed phenomenon using higher resolution, non-immunoassay based methods such as electron paramagnetic resonance (EPR; e.g. see [18]). Based on these observations, it is likely that repeat length- and temperature-dependent behaviour of polyglutamine repeats may be observable more generally with other polyQ proteins, such as those associated with spinocerebellar ataxias, spinal bulbar muscular atrophy (SBMA) and dentatorubral-pallidoluysian atrophy (DRPLA) [60]–[62]. Additionally, our studies highlight the importance of carefully controlling temperature in measuring levels of proteins comprising polyglutamine repeats, particularly where such assays involve the use of antibodies interrogating the polyQ repeat [13]. Although the precise mechanisms regulating N-terminal HTT conformation are only starting to be understood,the N17 region is subject to post-translational modifications (PTMs), including phosphorylation, acetylation, ubiquitination and SUMOylation which significantly alter HTT N-terminal fragment biological properties *in vitro* and in cells [17], [22], [23], [45], [63]. For example, mimicking the presence or absence of phosphorylation on specific N17 residues has a significant impact on HD-related pathology *in vivo* and can alter N-terminal HTT flexibility [20], [26]. Although the temperature- and polyQ-dependent conformational event reported here does not appear to be influenced by such mutations, the availability of rapid, robust, scalable assays sampling HTT conformational aspects in purified proteins of a defined nature (fragment length, polyQ length, presence of PTMs; e.g. see [16]) as well as relevant biological samples allows the interrogation of the role of conformational aspects of HTT in HD pathology, for instance to determine whether HTT conformational differences can explain variations in disease progression in patients with identical polyQ expansions, as well as enable drug discovery efforts aimed at modulating mutant HTT conformation through genetic or pharmacological means. In conclusion, we have demonstrated using different methodologies that the conformation of HTT proteins (studied either as recombinant proteins or as proteins expressed in cell lysates) can, when supported by orthogonal methods, be interrogated using appropriately controlled immunoassays, and that HTT protein structure may be sensitive to genetic (e.g., polyQ length) as well as non-genetic (e.g., temperature) factors. Although further structural studies are necessary, these data confirm previous studies suggesting that the N-terminal region of HTT protein is flexible and becomes less so upon polyQ expansion [26], and converge on the hypothesis that HTT protein conformation may be amenable to modulation. Finally, heterogeneity in disease manifestation amongst patients bearing the same polyglutamine expansion supports the possible existence of disease modifiers which may include events that affect HTT protein conformation, a hypothesis that can be tested with the assays presented here.

### Note added during revision

Following submission of this manuscript, a manuscript appeared illustrating largely comparable findings to those presented in this present report [64].

## Materials and Methods

### Plasmid constructs

cDNAs encoding N-terminal fragments (exon 1, N548) or full length human HTT bearing different polyQ lengths were obtained as follows. cDNAs encoding human HTT exon 1 fragments (Q16, Q39 or Q72) were synthesized (Genscript, Piscataway, NJ) and subcloned into pCDNA3.1 either with or without a C-terminal EGFP translational fusion. Mammalian expression constructs for cDNAs encoding human HTT N548 fragments (Q16, Q39 or Q72) or full length HTT (Q18, Q83) in pCDNA3.1 were reported previously [13].

### Protein expression and purification

Recombinant human proteins containing the N-terminal sequence of HTT with 548 amino acids (N548) and a polyQ repeat of different length were generated as previously described [13]. THRX-HTT exon 1-Q46 and Q25 were expressed by the previously used pET32a-HD46Q [18] and pET32a-HD25Q [65] parent constructs. Expression and subsequent lysis of cell pellets were performed as previously described [18]. Then, similarly to the previously described protocol, proteins were purified by centrifuging the lysates at 19,000×*g* for 30 min; incubating with nickel-nitrilotriacetic acid-agarose beads (Qiagen) on a rocker at 4°C for 1 hr; washing with several column volumes of 20 mm Tris-HCl, pH 8.0, 300 mm NaCl, 50 mm imidazole; and eluting with 20 mm Tris-HCl, pH 7.4, 300 mm NaCl, 250 mm imidazole. Following concentration of the proteins via Amicon Ultra-15 10,000 MWCO centrifugal filters (Millipore), proteins were re-diluted in 20 mm Tris, pH 7.4, then purified on a HiTrap Q XL column (GE Healthcare) with an AKTA FPLC system (Amersham Pharmacia Biotech), taking 1-ml fractions and using a phosphate-buffered saline gradient from 20 mm sodium phosphate, pH 7.4, 50 mm NaCl, to 20 mm sodium phosphate, pH 7.4, 1 m NaCl. For isolation of the THRX fusion partner, the fusion protein was cleaved with EK_max_ (Life Technologies, Grand Island, NY) at room temperature for 30 min. Then, 1 m urea was added to the resulting fragments, which were subsequently applied to nickel-nitrilotriacetic acid-agarose beads and briefly centrifuged in a tabletop Eppendorf 5415D centrifuge at 13,200 rpm. The supernatant was diluted 1∶10 in 20 mm, Tris pH 7.4, and further purified by FPLC as described for THRX-HTT exon 1 above.

### Cell lines

HEK293T cells were cultured in DMEM, 10% FBS, 1% Penicillin and Streptomycin (all from Gibco by Life Technologies) as per supplier's instructions. Cells were routinely transfected with plasmid constructs using Lipofectamine2000 (Life Technologies) as per manufacturer's instructions. Forty-eight hours later, transfection cells were harvested and lysed in lysis buffer (PBS, 0.4% Triton X-100) supplemented with 1X protease inhibitor cocktail (Roche).

### Antibodies

The MW1 antibody recognizing an epitope within the polyQ stretch of HTT was developed by Paul Patterson [27] and obtained from the Developmental Studies Hybridoma Bank developed under the auspices of the NICHD and maintained by The University of Iowa, Department of Biological Sciences, Iowa City, IA 52242. 2B7 antibody binds to the N17 region of HTT; its generation and characterization were described previously [32]. 4C9 antibody was raised against the human PRR region in exon1 of the HTT protein [66]. 3B5H10 antibody is specific for the polyQ stretch of HTT and was generated and characterized by Steve Finkbeiner [67], [68]. 2B7, 4C9 and MW1 antibodies were obtained from the CHDI Foundation (New York, NY). Antibodies MAB2166 and 3B5H10 were obtained from a commercial source (Millipore cat. n. MAB2166 and Sigma cat. n. P1874, respectively). Custom terbium cryptate and D2-fluorophore antibody labeling was performed by CisBio (Bagnols, France). Depending on the batch used, antibodies were cross-linked to 5 to 7 mol of terbium cryptate or D2-fluorophore per mole of antibody. Alexa-647 labeled 2B7 antibody was generated using the Alexa Fluor 647 Monoclonal Antibody Labeling Kit from Life Technologies (cat.n. A20186) as per manufacturer's instructions. Antibody against GAPDH was from Sigma (cat.n. G9545).

### Western blot assay and Coomassie staining

Total protein levels of lysates from HEK293T cells were quantified using the BCA Protein Assay kit (Novagen) as per manufacturer's protocol. 20 µg of total lysates were denatured at 95°C in 4X loading buffer (125 mM TrisHCl pH 6.8, 6% SDS, 4 M urea, 4 mM EDTA, 30% glycerol, 4% β-mercaptoethanol and Bromophenol blue) and loaded on NuPAGE 4-12% Bis-Tris Gel (Life Technologies). Proteins were transferred on PVDF membranes using the transfer apparatus from Life Technologies or Trans-Blot Turbo Transfer System from Biorad following manufacturer's protocol. Membranes were stained for 30 minutes in TBS, 0.1% Tween, 0.4% PFA, before blocking (1 hr) in TBS, 0.1% Tween, 5% non fat milk. Incubations with primary antibodies were performed overnight at 4°C. Incubations with secondary anti-mouse or -rabbit horseradish peroxidase conjugated antibodies were carried out for 1 hour at room temperature. Protein bands were detected using chemiluminescence substrate (ECL from Life Technologies). Similarly 0.25 µg or 2–5 µg of human recombinant HTT N548 proteins were loaded on NuPAGE 4–12% Bis-Tris Gel (Life Technologies) for Western blotting analysis and for fast coomassie-G250 staining respectively.

### TR-FRET assays

TR-FRET assays were performed as described previously [32]. In brief, 5 µl of each sample was transferred to a low volume 384 well plate (Greiner) in serial dilutions starting from a defined concentration (1–4 µg/µl for total lysates and 2 ng/µl for recombinant proteins). 1 µl of antibody cocktail was then added. Soluble HTT was measured with 2B7-Tb/MW1-D2, 2B7-Tb/3B5H10-D2, 2B7-Tb/4C9-D2 using 1 ng/µl of 2B7-Tb and 10 ng/µl of D2 labeled antibody. TR-FRET measurements were routinely performed following incubation for 1 hour at room temperature and following incubation for 1 hour or overnight at 4°C using an EnVision Reader (Perkin Elmer) following excitation at 320 nm (time delay 100 µsec, window 400 µsec, 100 flashes/well). Values were collected as the background subtracted ratio between fluorescence emission at 665 nm and 615 nm where the background signal corresponds to the ratio (665/615) measured for the antibodies in lysis buffer. The points in the graphs correspond to the averages of the background subtracted fluorescence ratio relative to the sample replicates and the bars represent the standard deviations among these replicates. The dilution points of each sample were fitted in a 4 parameters function that describes the curves. In each experiment, in order to obtain a unique value describing the temperature- and polyQ-dependent variation in TR-FRET signal, the ratio between the maximum fluorescence value of the fitted curve at 4°C and the maximum fluorescence value of the fitted curve at room temperature was calculated. The columns in the presented graphs correspond to the averages of such ratios obtained in different TR-FRET experiments. The bars represent the standard deviations among values obtained in different experiments. The ratios were also expressed as relative fold differences with respect to ratios obtained for wild type HTT protein (Q16). Significance was calculated using the one-way ANOVA test and Bonferroni's multiple comparison post-test (* = p<0.05; ** = p<0.01; *** = p<0.005; **** = p<0.001).

### CD study

CD was performed using a Jasco 815 spectropolarimeter (Jasco Inc., Easton, MD). Temperature was regulated by a Jasco PFD-425S Peltier type FDCD attachment connected to a PolyScience recirculator (PolyScience, Niles, IL). For spectra at each temperature, measurements were taken every 1 nm from 200 to 260 nm, scanning at 50 nm/min and an averaging time of 1 sec. For spectra spanning from 190 to 260 nm, protein was eluted into 20 mm sodium phosphate, pH 7.4, no salt, via a PD-10 column (GE Healthcare) and CD measurements taken with the parameters denoted above. Ten scans, or twelve scans for 190-to-260-nm readings, were averaged for each sample spectrum; background spectra were obtained by averaging twenty scans and the appropriate ones were subtracted from the respective sample spectra. Spectra were smoothed by the Savitsky-Golay algorithm. Single-wavelength readings at 222 nm were obtained at −10°C, 0°C, 4°C, 10°C, 20°C, 30°C, and 37°C. In each case, ellipticity was measured every 1 sec for 300 sec; the 301 readings for each sample at each temperature were averaged. The number of amino acids in each htt exon 1 sample to experience a change in helicity from at −10°C to 37°C was estimated using a previously developed helix-coil transition model, which gives the change in fraction of helicity (Δ*f*_H_) by the following equation [36], [69]:
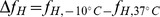
where *f*_H_ at each temperature is given as follows:
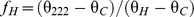
where θ_222_ describes the helix coil transition in exon-1, obtained from the difference between the observed MRE at 222 nm of the fusion protein at the given temperature and the product of the MRE at 222 nm of Trx alone at the same temperature and the fraction of residues comprised by Trx in that particular construct, and 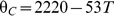
(2a)and 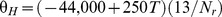
(2b)given that *T* is the temperature in °C and *N*_r_ is the number of residues. For computation using DICHROWEB, which requires input spectral data with the lowest wavelength point at 190 nm or lower, spectral data sets collected for the Q25 and Q46 constructs in salt-free buffer at −10°C and 37°C were entered and analyzed with the SELCON3 and CONTIN analysis programmes, using the reference set SP175 [37], [38]. Programs were chosen to provide best fit of the data. Values resulting from CONTIN analysis are those of the closest matching solution with all proteins.
