# Correction: Polyglutamine- and Temperature-Dependent Conformational Rigidity in Mutant Huntingtin Revealed by Immunoassays and Circular Dichroism Spectroscopy

**DOI:** 10.1371/journal.pone.0129986

**Published:** 2015-06-03

**Authors:** The PLOS ONE Staff

In the Methods section, Equation 2b is incorrect. The correct equation is: θ_H_ = (–44,000 + 250*T*)(1–3/*N*_r_)
